# A tool for developing an automatic insect identification system based on wing outlines

**DOI:** 10.1038/srep12786

**Published:** 2015-08-07

**Authors:** He-Ping Yang, Chun-Sen Ma, Hui Wen, Qing-Bin Zhan, Xin-Li Wang

**Affiliations:** 1 Department of Entomology, China Agricultural University, Beijing, China; 2 Climate Change Biology Research Group, State Key Laboratory for Biology of Plant Diseases and Insect Pests, Institute of Plant Protection, Chinese Academy of Agricultural Sciences, Beijing, China; 3 Nanjing Forest Police College, Nanjing, China

## Abstract

For some insect groups, wing outline is an important character for species identification. We have constructed a program as the integral part of an automated system to identify insects based on wing outlines (DAIIS). This program includes two main functions: (1) outline digitization and Elliptic Fourier transformation and (2) classifier model training by pattern recognition of support vector machines and model validation. To demonstrate the utility of this program, a sample of 120 owlflies (Neuroptera: Ascalaphidae) was split into training and validation sets. After training, the sample was sorted into seven species using this tool. In five repeated experiments, the mean accuracy for identification of each species ranged from 90% to 98%. The accuracy increased to 99% when the samples were first divided into two groups based on features of their compound eyes. DAIIS can therefore be a useful tool for developing a system of automated insect identification.

Correct insect identification is critically important in entomological research, pest control, and insect-resource utilization. However, to identify an unknown species using traditional methods is very time consuming due to the vast number of insect species that must be identified and limited taxonomist resources^1^,^2^. This problem has been recognized since the end of the last century, and computer-based recognition systems have been developed to identify insect species automatically^3^. For example, the “Digital Automated Identification System (DAISY)”^4^,^5^,^6^ was developed to automatically identify ichneumonid wasps on the basis of their wing features^3^, and this program was also used to identify biting midges and moths^6^. The “Species Identified Automatically (SPIDA)” system was originally designed to distinguish between 121 species of Australian spiders^7^ and has been successfully tested for identifying wasps and bees using their wing characteristics^5^. In addition, the “Automatic Bee Identification System (ABIS)” was developed to identify bees by analyzing images of wing veins^8^.

All of these automatic identification systems involve two key processes: extracting and digitizing the taxonomic features and constructing a classifier model for identification.

First, a recognition feature must be selected. The feature must be not only stable and distinguishable but also extractable and quantifiable by computer. The feature “color” was successfully used to identify moths and butterflies^9^, but its accuracy was limited by specimens fading with time or color shifts under different illumination. By contrast, the outlines of body parts, such as wings, are stable and diverse but have not been frequently used in conventional taxonomy due to difficulties with lexical descriptions. Therefore, wing outlines represent a potential new characteristic for computer-based insect identification.

In recent decades, software has been developed to digitize morphological characteristics and conduct consequent statistical analysis, such as principal component analysis and canonical correlation analysis, to establish the phylogenic relations of species. Examples of this software include TPSDig^10^, DrawWing^11^,^12^,^13^ and SHAPE^14^,^15^, which are based on modern digitalizing methods, such as ‘Landmark’, ‘Semilandmark’^10^,^11^ and Elliptic Fourier Descriptors (EFDs)^16^,^17^,^18^,^19^,^20^. In particular, for the extraction and digitization of outline characters, the Elliptic Fourier algorithm has the advantages of being able to reconstruct outlines, eliminate errors in orientation caused by interference, size images and trace the starting point of an original image^21^,^22^,^23^. However, no classifier models or automatic identification functions were included in these software packages.

The support-vector-machines algorithm (SVMs) was invented by Vapnik^24^ and has since become a popular method for pattern recognition^25^,^26^. SVMs have also been used for insect identification^9^,^27^. Comparative studies in which SVMs and other 4 methods (Bayes, instance-based learning, decision trees and random forests) were used to identify 35 species of moths suggested that the SVMs algorithm produced the most accurate results^27^.

Although progress has been made in automatic identification systems for insects, two inadequacies remain. First, some systems, such as ABIS^8^, obtain high accuracy of identification for limited insect species but have very narrow functionality. When these types of system are tested with new morphological insect data, the source code for these computer programs must be rewritten, or the outputs must be interpreted in a complex manner, restricting widespread application for most insect taxonomic users and limiting further improvements. Second, some systems, such as DAISY^4^ and SPIDA^7^, can be used to identify different taxonomic groups of insects, but the accuracy is not sufficient due to high variations during processes of digitizing morphological features and insufficient powerful classifiers which only can search local optimizing solutions rather than the global optimizing solutions during computation.

To address these issues, we aimed to build an ideal user-friendly tool for developing an automatic insect identification system based on wing outlines. This program only requires a user to load a minimum set of images of the wing spread perfectly on a horizontal plane into the DAIIS program (Tool for Developing an Automatic Insect Identification System). The system can automatically extract the wing outline as a diagnostic feature. The outline will then be transformed into EFDs, i.e., a set of coefficient data. Some of the data will be used as training dataset to construct the recognition model, i.e., classify automatically with an SVM algorithm in our system. The remaining data will be used as candidate samples for identification. We have designed the system to give the user an accuracy measure, which is partly reliant on the quality and quantity of the input data. In addition, DAIIS can be modified and extended easily by broad taxonomic users who do not have extensive computer programing experience.

Most adult insects have wings that are frequently used in taxonomy. However, the detailed characteristics of veins and spots on wings require recognition and measurement by well-trained experts; thus, these features are difficult for general users to use. Wing outlines are stable and diverse, making this factor useful for insect identification^28^. Owlflies (Insecta: Neuroptera: Ascalaphidae) are similar to dragonflies, and the wing outlines of this family are diverse and provide ideal identification for some genera and species. In this study, we used the DAIIS and 120 owlfly specimens belonging to seven species to train an owlfly classifier and assess its accuracy.

## Results

### Character extraction and description of wing outlines

**Figure 1:**
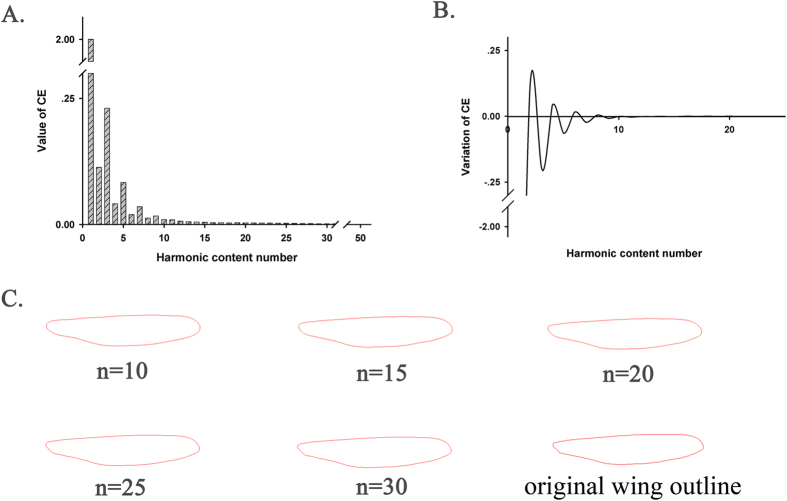
Relationship between the descriptive precision of a reconstructed wing outline and the harmonic content number. (**A**) A histogram demonstrates that the values of the Elliptic Fourier descriptor (CE) change with increasing harmonic content numbers. (**B**) A wave line demonstrates that the variation in CE declines with increasing harmonic content numbers. (**C**) Wing outlines reconstructed from the first 10, 15, 20, 25, and 30 harmonics and the original outline.

According to the descriptor formula (5), as the harmonic content number increases, the variation of the Elliptic Fourier descriptor (CE)^29^ (i.e., value of CE calculated with the present harmonic content number minus the value of CE computed with the previous harmonic content number) becomes smaller and eventually negligible. The *i*th harmonic is generally used to represent the maximum harmonic content number needed in the Elliptic Fourier transformation. Our results indicate that the variation in the CE decreased as harmonic content numbers increased and became nearly zero after the 15th harmonic (Fig. 1B). Based on observations and comparisons of hundreds of owlfly specimens, we found that the projecting axillary angle was highly diversified in some owlfly species. Thus, the detailed shape around the base of the owlfly wing was treated as a key recognizable characteristic for different species. Accordingly, the reconstructed outline around the base of the wing should be carefully considered in determining the final harmonic content numbers. We reconstructed wing outlines with a loop from the 10th to the 50th harmonic with a step size of five and found clear outlines around the wing base up to the 30th harmonic (Fig. 1C). Thus, the first 30 harmonics were considered appropriate for approximating the outlines of owlfly wings. We obtained 120 EF coefficients (four coefficients per harmonic) for each wing outline. All coefficients were normalized; 117 coefficients were recognition features, and the remaining 3 were constants (*a*_*1*_ = 1, *b*_*1*_ = 0, and *c*_*1*_ = 0).

**Figure 2:**
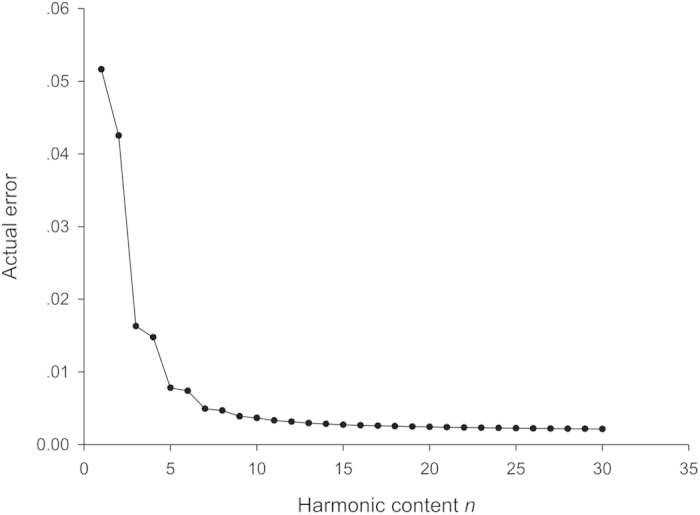
Actual error versus harmonic content number.

The actual error of the reconstructed outlines compared with the original, *ε*_*n*_, decreased rapidly as the harmonic content number increased (Fig. 2). Our results indicate that the coefficients of the first 30 harmonics can accurately approximate the original outline.

### Results of automatic owlflies identification

We obtained high accuracy with five cross-validations^30^ (a method for training the model) and an independent validation (predicted with the test dataset and model) of the identification model for owlflies (Table 1). The mean accuracy of the cross-validation (CV) and independent validation was as high as 97.7% and 94.5%, respectively. When a real identification was conducted by using our model and the independent dataset (not used in training), the lowest identification accuracy of 87% was observed for the species *Protidricerus elwesii*, and the highest accuracy of 100% was observed for three species, *Ascalohybris subjacens*, *Libelloides sibiricus*, and *Protidricerus japonicus* (Table 2).

### Misidentification and improvement

**Figure 3:**
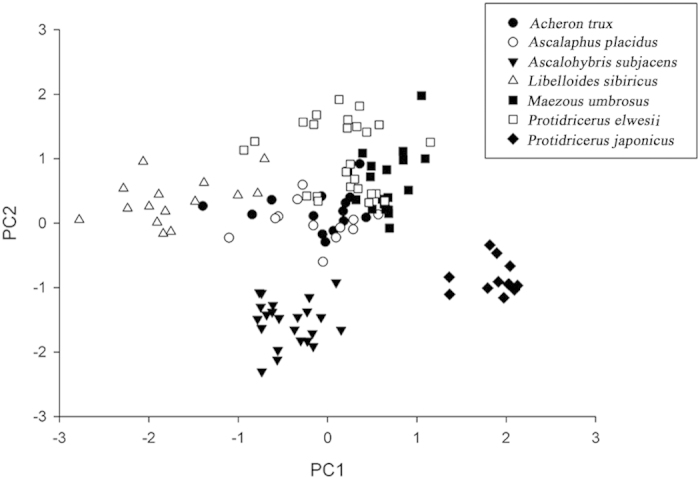
The results of the principal component analysis illustrate the landscape of misidentification between all 7 owlflies species using wing outlines. PC1 and PC2 are the first and second principal components, respectively.

The detailed misidentification rates for seven species of Ascalaphidae are shown in Table 3. *P. elwesii* had the greatest likelihood (8.33%) of being confused with *Maezous umbrosus*. *Ascalaphus placidus* was also relatively easily confused with *L. sibiricus* (misidentification rate = 6.67%). To illustrate the landscape of misidentification among all 7 species in this study, we conducted a principal component analysis (PCA) and used the first two principal components to construct a scattered graph (Fig. 3). The wing outlines are overlapped among 4 species, i.e., *Acheron trux*, *M. umbrosus*, *P. elwesii*, and *A. placidus*, but are clearly separated from those of *A. subjacens* and *P. japonicus*. Thus, improving the identification accuracy of these three owlflies species is difficult when using only wing outlines.

**Figure 4:**
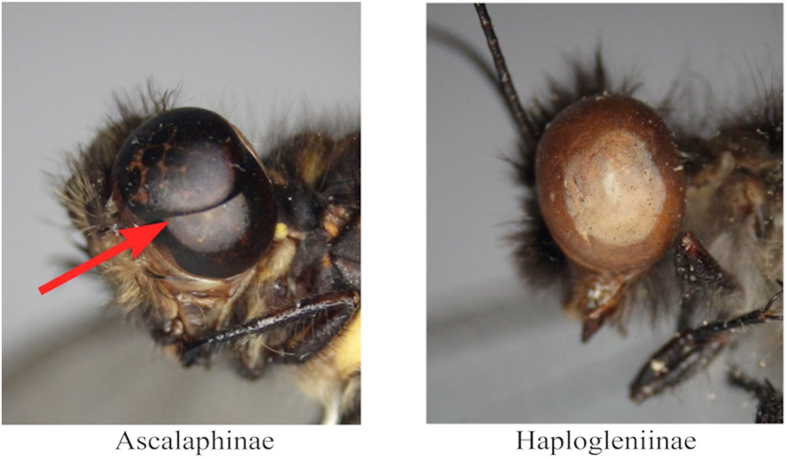
Compound eye of two subfamilies of owlflies, Ascalaphinae and Haplogleniinae.

However, owlflies (Ascalaphidae) are divided into two subfamilies, Ascalaphinae and Haplogleniinae, based on significant features of their compound eyes, which can be distinguished with the human eye. Each compound eye of Ascalaphinae contains a visible furrow that divides the complete eye into two parts, whereas the eye of Haplogleniinae is a complete compound eye without such a division mark (Fig. 4). Of the seven species, *P. elwesii* and *P. Protidricerus* belong to Haplogleniinae, and the other five species belong to Ascalaphinae. Thus, compound eyes enable the seven species to be separated into two groups, and all species can be identified correctly with wing outlines using two separate classification models trained for each subfamily. Using this approach, the identification accuracy of *A. placidus* was over 96%, and the accuracies of the other six species were 100%. The mean identification accuracy of all specimens was 99% (Tables 4 and 5).

## Discussion

We developed DAIIS as a simple tool to help broad users who do not have extensive knowledge of algorithms and programing to develop their own automatic identification systems based on outlines of body parts. Users must simply download our software package and copy their outline images to the “doTrain” and “doPre” folders to train the identification model and subsequently perform the actual identification, respectively. Users can therefore focus on their own area of expertise rather than designing the algorithm and programing. To our knowledge, this is the first tool for developing automatic identification systems for any species using SVMs and EF coefficients of body part outlines.

To reduce operational error when capturing and processing wing images, we developed a set of guidelines (cutting the wing image from a specimen) or apparatus (white background under fixed lights) for image capture and processing. To reconstruct a very accurate outline with Elliptic Fourier transformation, we determined the first 30 harmonics to produce 120 coefficients. To minimize the impact of variance on image size, rotating direction and starting point to trace an outline, we conducted standard transformations of these coefficients, which contributed to our high identification accuracy.

The algorithms used here contributed to successful identification based on the outlines. The number of samples used in model training was limited, but the number of features for each sample was as high as 117. The SVM was shown to be a useful algorithm for situations with small sample sizes but high dimensional space. Furthermore, we used the genetic algorithm to optimize the parameters in the radial basis kernel function (RBF) of the SVM, which searched the global optimal solution in large search spaces more efficiently than other competing algorithms, such as the grid algorithm^31^.

Although we have not explicitly tested DAIIS on data other than owlflies wings, we see no reason why this system could not be extended to adapt for other insect groups with rich diversity in wing outlines, other body parts and even wing spots. Furthermore, DAIIS can be extended to automatically identify insects, other groups of organisms or even any other physical objects based on more recognizable characteristics, such as size, color, veins of wings etc., by developing corresponding digitalized algorithms for these features.

In the present study, we developed a package in MATLAB and made it available for download as a standalone executable application (.exe). However, our package can also be delivered into an application programing interface (API) as a library file (.dll), enabling its use in other programs developed in Java, C++, Visual Basic, etc. Therefore, our package is highly flexible and expandable for further secondary development. The program and test image data can be downloaded from http://www.au2id.cn/package.rar and should be run in Windows XP in a Matlab environment.

## Materials and Methods

### Insect materials

We collected 120 specimens belonging to seven species of owlflies from the Insects Collections of China Agricultural University (ICCAU), Beijing, China. The list of species and species IDs is shown in Table 6. Only right forewings were used as a demonstration for this study.

### System procedure

**Figure 5:**
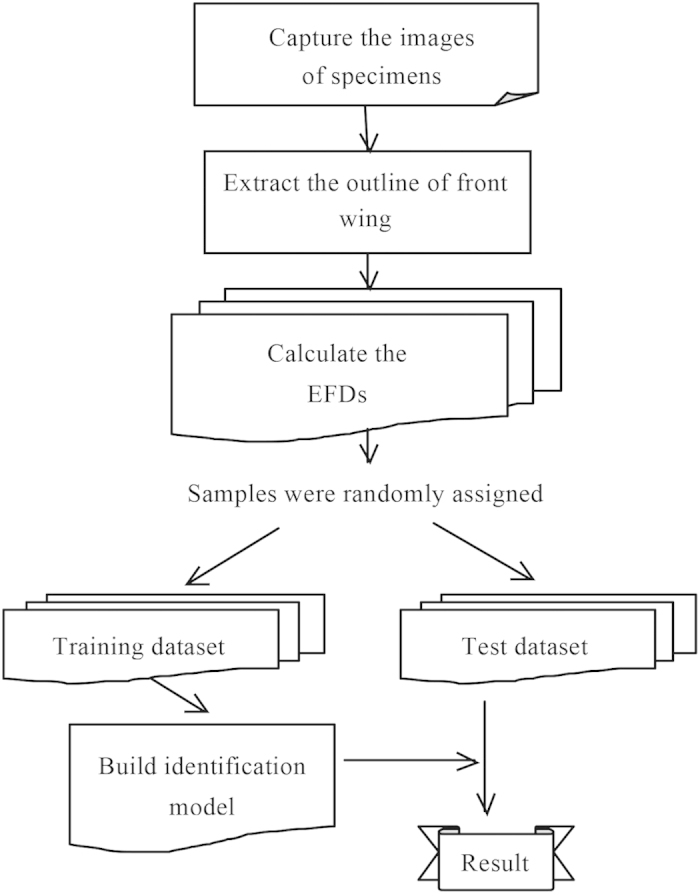
Flowchart of the automated identification system.

The system primarily includes two subsystems (Fig. 5). The first subsystem is the identification model generator, which builds the identification model with SVMs and a training dataset. The second subsystem is the model validator, which calculates the identification accuracy by comparing the model output with test data. The training and test samples were identified by a taxonomist. All data were randomly divided into the training and test datasets at a 1:1 ratio. After a valid identification model is established, users can apply our system to identify an unknown specimen. In this paper, we describe the procedures to implement those functions step by step.

### Capturing the wing image

**Figure 6:**
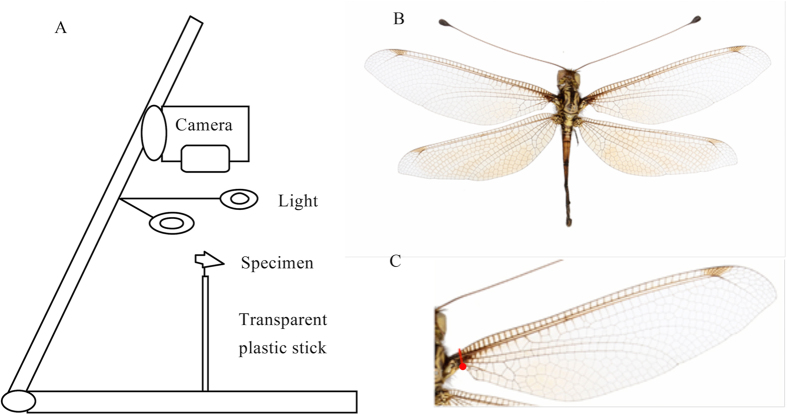
Capturing images of owlflies specimens and their wings. (**A**) Schematic of apparatus used to photograph owlflies specimens. (**B**) Complete image of an owlflies specimen. (**C**) Position where the wing is cut from the specimen image.

Owlfly images were captured with a digital camera (Canon 60D, 60 mm lens) fixed to a copy board that had fill lights on both sides (250 W each) and a white foam sheet at the base. To eliminate the shadow of the specimen due to the fill lights (for easier image capture), a transparent 2-mm-diameter, 100-mm-long plastic stick was fixed to the foam and held at the far end of a needle that was inserted into the specimen (Fig. 6A). The wings of the specimen were set to be as horizontal as possible prior to image capture, and the photographs were saved as. jpg files.

A standard method was used to obtain the wing shapes from the above images. The background of the image was easily removed using Photoshop (version 12.0.4). To ensure that a perfect wing shape was obtained, especially for the base part of the wing, the first inflection point from the lower edge of the base of the wing was fixed as the starting point for cutting the wing image. Then, the wing was cut perpendicularly to the upper edge of the wing (Costa, C) (Fig. 6C). Finally, the image of the wing was copied and saved to a new. bmp image file. The wing was adjusted to a unified direction by turning the base of the wing to the left and rotating the lower edge of the wing (Costa, C) horizontally.

For individual specimens in which the wing edge was slightly cracked, the wing outline image was repaired manually by drawing a connecting line in Photoshop with the Digitizing Tablet (type Wacom Bamboo CTL-470).

### Obtaining wing outline

**Figure 7:**
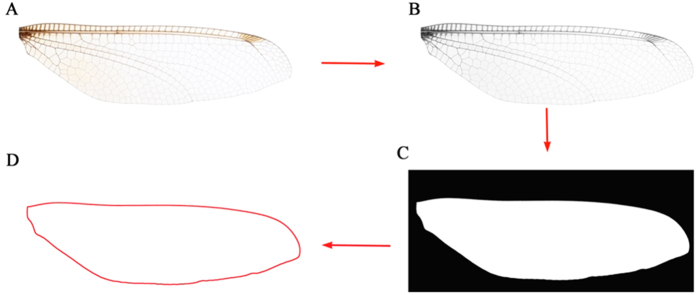
Image processing steps to obtain a wing outline image from a. bmp wing image. (**A**) Original. bmp image. (**B**) Eight-bit grayscale image. (**C**) Binary image after noise reduction, erosion, dilation, and hole filling. (**D**) Image of the wing outline.

We developed a function module with MATLAB to automatically transform the above wing image into a pure white-black wing outline image. By running this module, the. bmp wing image can be transformed into an eight-bit grayscale image (256 levels of gray) and then converted into a binary image. Finally, the outline was obtained after noise reduction, erosion, dilation, and hole filling (Fig. 7).

### Digitalizing wing outline

To approximate a wing outline with an irregular shape, we used a piecewise-linear number sequence generated by a chain encode^32^ that supplies either a 4-directional chain code or 8-directional chain code. We selected the 8-directional chain code, which consists of eight standardized line segments^33^ for more accurate approximation. In this sequence, the angle π/4 (as measured counterclockwise from the *x* axis of an *x-y* coordinate system) was multiplied by a number between 0 and 7 to determine the orientation. If we assume that the length between adjacent points (*i* − 1) and *i* is 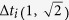, then the length *t*_*p*_ from the starting point to point *p* can be calculated by equation (1), and the *x* and *y* coordinates of point *p* can be calculated by equation (2).


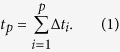



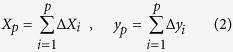


With this method, the wing outline was traced and converted to continuous *x* and *y* coordinates.

We used EF to transform these coordinates into Elliptic Fourier coefficients. The series expansions of the Elliptic Fourier transform for the *x* and *y* coordinates of the complete outline are shown in equations (3) and (4), where *a*_*n*_, *b*_*n*_, *c*_*n*_ and *d*_*n*_ are the Elliptic Fourier coefficients for the *n*th harmonic.


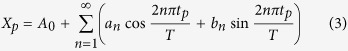



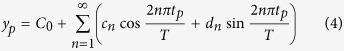


The Elliptic Fourier coefficients were used to reconstruct outlines. The variation between the reconstructed and original outline depends on the number of harmonics used. More harmonics would lead to a better-reconstructed outline. However, Elliptic Fourier analysis becomes more complicated and time consuming as the harmonic content number increases. Thus, appropriate numbers of harmonic content should be determined to compromise between computation speed and clarity of the reconstructed characteristics.

Here, we define the invariant descriptors of the Elliptic Fourier (CE) as a flow expression given by Granlund^29^:


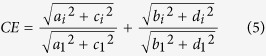


where *a*_*i*_, *b*_*i*_, *c*_*i*_ and *d*_*i*_ are the Elliptic Fourier coefficients for harmonic *i*.

Here, we set 30 as the default harmonic content number in DAIIS. If a more accurate reconstructed outline is needed, the harmonic content number can be modified in the file “config.txt”.

Then, we calculated the *x* and *y* coordinates of points on the reconstructed outline based on the cumulative contributions of the first 30 harmonics. Next, the reconstruction error was calculated according to formula (6).


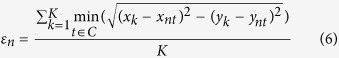


where *ε*_*n*_ is the error between the reconstruction and original outlines based on the first *n* harmonics, *K* is the number of the points on the original outline, (*x*_*k*_, *y*_*k*_) are the coordinates for point *k* on the original outline, and (*x*_*nt*_, *y*_*nt*_) are the coordinates for point *t* of the reconstruction.

To minimize the impacts of image size, rotation, and starting point of the outline trace, the EF coefficients were normalized using the method of Kuhl and Giardina^33^. After normalization, the first three coefficients of the first harmonic are constants for *a*_*1*_ = 1, *b*_*1*_ = 0, and *c*_*1*_ = 0.

With this procedure, the black-white image of wing outline can be automatically transformed into the EF coefficients.

### Training and testing the identification model

All of the EF coefficients of wing outlines from each of the seven owlflies species were divided into a training dataset and test dataset at a 1:1 ratio. The C-Support Vector Classification (C-SVC) with a radial basis kernel function (RBF), which is one of the most common types of SVMs, was used for building the identification model^34^. The kernel function was used to search for the optimal resolution in a nonlinear higher-dimensional space, in which the genetic algorithm (GA)^35^ was applied to optimize the parameters of the kernel based on the analogous theories of Darwinian natural selection and genetics in biological systems. With this procedure, a primary optimal identification model was ready for validation.

To validate our model for owlflies more efficiently with limited samples, we conducted 5 randomized runs of model training and validation. For each run, all datasets of wing outlines were divided randomly and equally into a training dataset and a test dataset. To obtain the optimal identification model, we adopted a cross-validation (CV) approach to calculate the CV values for optimizing the model parameters. To demonstrate the accuracy of the identification model, we input the test dataset into the model, predicted the species, and then calculated the proportion of prediction successes based on our experience of owlfly taxonomy. After 5 randomized runs, we calculated the misidentification rates using formula (7):


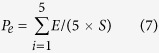


where P_e_ is the rate of species A being misidentified as species B in 5 randomized runs, E is the number of species A misidentified as species B for each randomized run, and *S* is the entire sample size.

To demonstrate the landscape of misidentification between all 120 specimens belonging to 7 species in this study, we conducted a principal component analysis and drew a scatter graph using the first two principal components, which contributed 88% in all variations.

All of the model training and identification with complex and professional programing source codes was packaged into an executable file named “dotrain.exe”.

### Using the automatic identification system

To use our system to generate a user’s own identification model and conduct further identification work, a user must copy images of wing outlines on a white background to the folder “doTrain” and then double-click the file named “doTrain.exe” in the same folder. The accuracy of the model is shown in a command prompt. To identify an unknown specimen, a user must move the image of the specimen into the folder “doPre” and then double-click the file “doPre.exe” in the folder to obtain the identification results in the command prompt.

## Additional Information

**How to cite this article**: Yang, H.-P. *et al.* A tool for developing an automatic insect identification system based on wing outlines. *Sci. Rep.* **5**, 12786; doi: 10.1038/srep12786 (2015).

## Figures and Tables

**Table 1:** Accuracy of the trained identification model derived from the five cross-validations (CV) and the independent validation.

Run	CV^*^ (%)	Accuracy^*^ (%)
1	98.4	89.655
2	96.8	94.828
3	98.4	94.828
4	96.8	98.276
5	98.387	94.828
mean	97.742	94.486

CV^*^ is the optimal accuracy with the cross-validation training for each run.

Accuracy^*^ is the actual accuracy of independent validation with test data in each run.

**Table 2:** Actual identification accuracy for each owlflies species.

Species	Accuracy (%)
*Acheron trux*	91.4
*Ascalohybris subjacens*	100
*Libelloides sibiricus*	100
*Maezous umbrosus*	95.6
*Protidricerus elwesi*	86.7
*Protidricerus japonicus*	100
*Ascalaphus placidus*	90.0

**Table 3:** The misidentification rates of the model for each owlflies species.

Actual species	Improperly identified species	Identification error rate(%)
*Acheron trux*	*Maezous umbrosus*	2.86
*Acheron trux*	*Protidricerus elwesi*	2.86
*Acheron trux*	*Ascalohybris subjacens*	2.86
*Maezous umbrosus*	*Protidricerus elwesi*	4.44
*Protidricerus elwesii*	*Maezous umbrosus*	8.33
*Ascalaphus placidus*	*Acheron trux*	3.33
*Ascalaphus placidus*	*Ascalohybris subjacens*	3.33
*Ascalaphus placidus*	*Libelloides sibiricus*	6.67
*Ascalaphus placidus*	*Maezous umbrosus*	3.33

Note: the misidentification rates for other species not listed in the table are zero.

**Table 4:** The improved accuracy of the trained identification model derived from the five cross-validations (CV) and independent validation after dividing all species into two groups based on the compound eyes feature.

Run	CV^*^ (%)	Accuracy^*^ (%)
1	97.67	100
2	100	100
3	100	95
4	100	100
5	97.67	100
mean	99.07	99

CV* is the optimal accuracy with the cross-validation training for each run.

Accuracy* is the actual accuracy of independent validation with test data in each run.

**Table 5:** The improved identification accuracy for each owlfly species after dividing all species into two groups based on the compound eyes feature.

Species	Accuracy (%)
*Acheron trux*	100
*Ascalohybris subjacens*	100
*Libelloides sibiricus*	100
*Maezous umbrosus*	100
*Protidricerus elwesii*	100
*Protidricerus japonicus*	100
*Ascalaphus placidus*	96.67

**Table 6:** Species and specimens of owlflies used for modeling and validation.

Species ID	Species	Sample size	Collection location
1	*Acheron trux*	14	Zhejiang, Fujian, Henan
2	*Ascalohybris subjacens*	23	Zhejiang, Fujian, Anhui, Jiangsu, Henan
3	*Libelloides sibiricus*	15	Beijing, Henan, Gansu, Shanxi, Liaoning
4	*Maezous umbrosus*	19	Guangxi, Yunnan, Henan, Hubei
5	*Protidricerus elwesii*	25	Zhejiang, Fujian, Guangxi, Tibet
6	*Protidricerus japonicus*	12	Beijing, Hebei, Henan, Gansu
7	*Ascalaphus placidus*	12	Zhejiang, Fujian, Anhui, Jiangsu

Notes: All specimens examined are deposited in the Insect Collections of China Agricultural University (ICCAU), Beijing, China.
